# Analysis of movements in tooth removal procedures using robot technology

**DOI:** 10.1371/journal.pone.0285503

**Published:** 2023-05-18

**Authors:** Tom van Riet, Willem de Graaf, Jan de Lange, Jens Kober

**Affiliations:** 1 Department of Oral and Maxillofacial Surgery, Amsterdam University Medical Center (AUMC), University of Amsterdam, Amsterdam, The Netherlands; 2 Academic Center for Dentistry Amsterdam (ACTA), University of Amsterdam, Amsterdam, The Netherlands; 3 Department of Cognitive Robotics, Mechanical, Maritime and Materials Engineering (3ME), Delft University of Technology, Delft, The Netherlands; Medical University of South Carolina, UNITED STATES

## Abstract

Being one of the oldest en most frequently performed invasive procedures; the lack of scientific progress of tooth removal procedures is impressive. This has most likely to do with technical limitations in measuring different aspects of these keyhole procedures. The goal of this study is to accurately capture the full range of motions during tooth removal as well as angular velocities in clinically relevant directions. An *ex vivo* measuring setup was designed consisting of, amongst others, a compliant robot arm. To match clinical conditions as closely as possible, fresh-frozen cadavers were used as well as regular dental forceps mounted on the robot’s end-effector. Data on 110 successful tooth removal experiments are presented in a descriptive manner. Rotation around the longitudinal axis of the tooth seems to be most dominant both in range of motion as in angular velocity. Buccopalatal and buccolingual movements are more pronounced in the dorsal region of both upper and lower jaw. This study quantifies an order of magnitude regarding ranges of motion and angular velocities in tooth removal procedures. Improved understanding of these complex procedures could aid in the development of evidence-based educational material.

## Introduction

In 1934, George Christiansen simplified tooth removal as removing a calcified substance from a bony socket lined by a fibrous membrane [1]. In his detailed paper, he provided expert instructions on what ideal movements in tooth removal should look like. In 1952, expert opinions on movements strategies from ‘authorities in the field of exodontia’ were summarized by Donald Kitzis [2]. Expert opinions, such as the aforementioned, regarding optimal tooth removal strategies lack, to date, a strong scientific background. This lack of scientific development is remarkable, since tooth removal is one of the most common and oldest surgical procedures worldwide.

In contrast to movement patterns, some literature is available in which forces exerted during tooth removal are measured and analyzed. Scientific attempts to objectify these forces, however, are often restricted in their design. They are either limited to a small selection of teeth *in vivo* [3,4], animal studies [5] or measured in an laboratory setting using a single tooth [6]. A scientific gap seems to exists in our knowledge of tooth removal [7]. In an effort to bridge this gap, the authors recently reported on forces and torques measured in experiments on fresh frozen cadavers [8]. The lack of scientific understanding of tooth removal has serious consequences for the education of dental students and most previous work in this field state to do so for educational reasons [6,9]. It is well known that students often feel unprepared before performing their first tooth removal on patients [10]. Preclinical training models are largely absent and, if used, rarely valued as representative [10]. Up until today, direct practice on patients, without significant preclinical training, is the most widely used training modality. However, in well-developed countries where preventive dentistry prevails, the opportunities to practice these procedures on patients are reducing. This situation potentially leads to less confident young dentists and more unnecessary referrals to oral and maxillofacial surgeons [11].

To benefit, amongst others, the development of new educational material, it is necessary to improve our knowledge of these complex procedures. Previous research aimed at analyzing forces in tooth removal, but research initiatives analyzing motion patterns are missing in literature [7]. The purpose of this study is to capture the full range of motions and angular velocities in a series of tooth removal experiments on fresh frozen cadavers. As explained in previous work describing a ‘proof of concept’, we hypothesized that robot technology will allow us to record high-frequency and high-resolution data of movements in tooth extraction [9]. Results will be presented in a descriptive manner and recommendations for future research will be discussed.

## Materials and methods

### Overview of the experiment

To capture movements during tooth removal in a reliable manner, an *ex vivo* measuring setup was considered most valuable, which is explained in detail in previous work [9]. Fresh frozen cadavers were obtained from the clinical anatomy and embryology section of the department of medical biology of the Amsterdam university medical center (Amsterdam UMC). The donation process was in accordance with Dutch legislation and the regulations of the medical ethical committee of the Amsterdam UMC. No separate approval was necessary by the medical ethical committee for anatomical studies according to local regulations. The authors state that every effort was made to follow all local and international ethical guidelines and laws that pertain to the use of human cadaveric donors in anatomical research [12].

**Fig 1:**
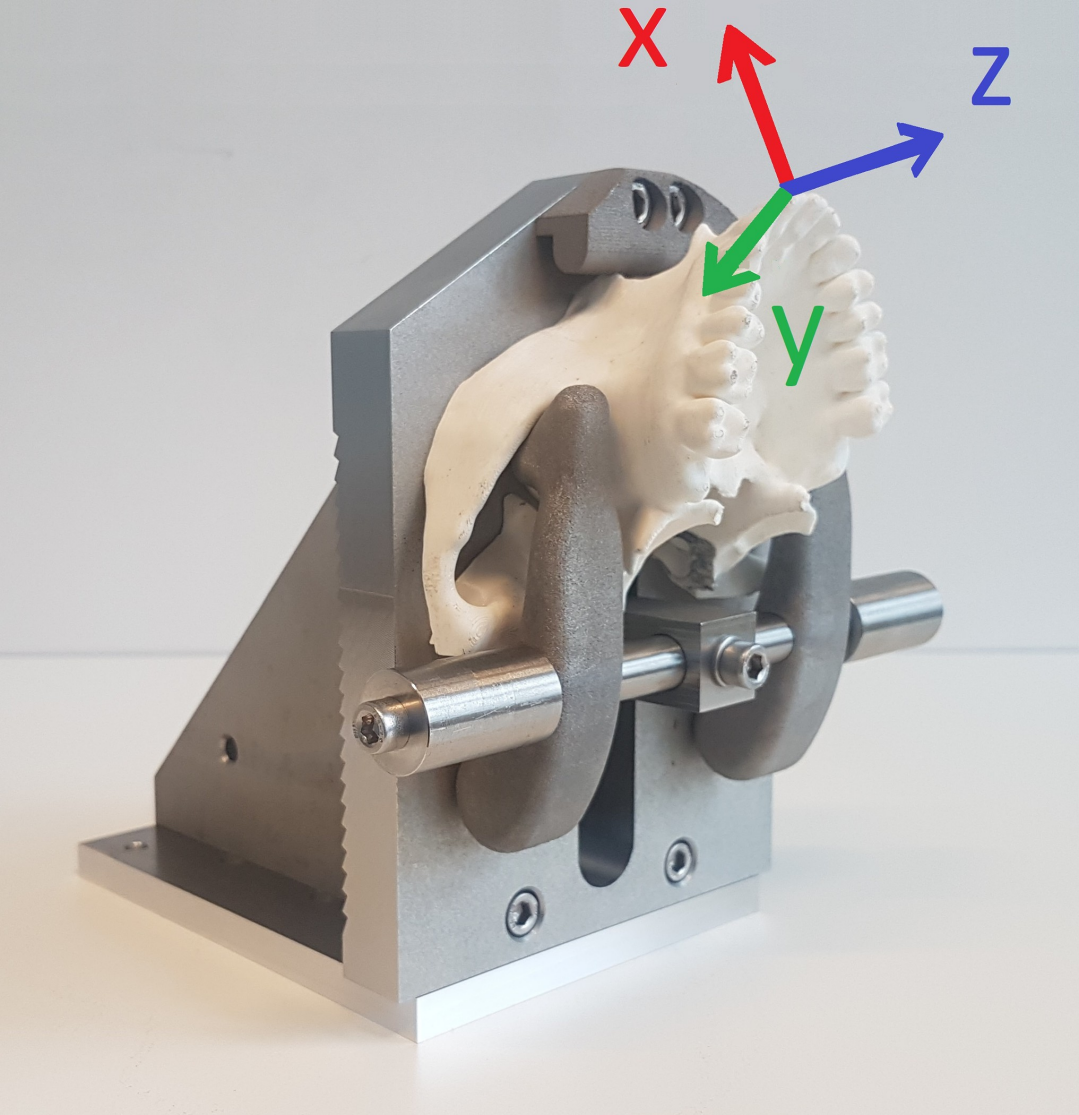
Upper jaw holding device. The white plastic model represents the reduced shape of the cadaveric upper jaw as prepared by the anatomical laboratorian. The reference frame is presented in 3 colored arrows. The X-axis (red arrow) represents the buccopalatal of buccolingual axis. The Y-axis (green arrow) represents the mesiodistal axis. The Z-axis (blue axis) represents the longitudinal axis.

**Fig 2:**
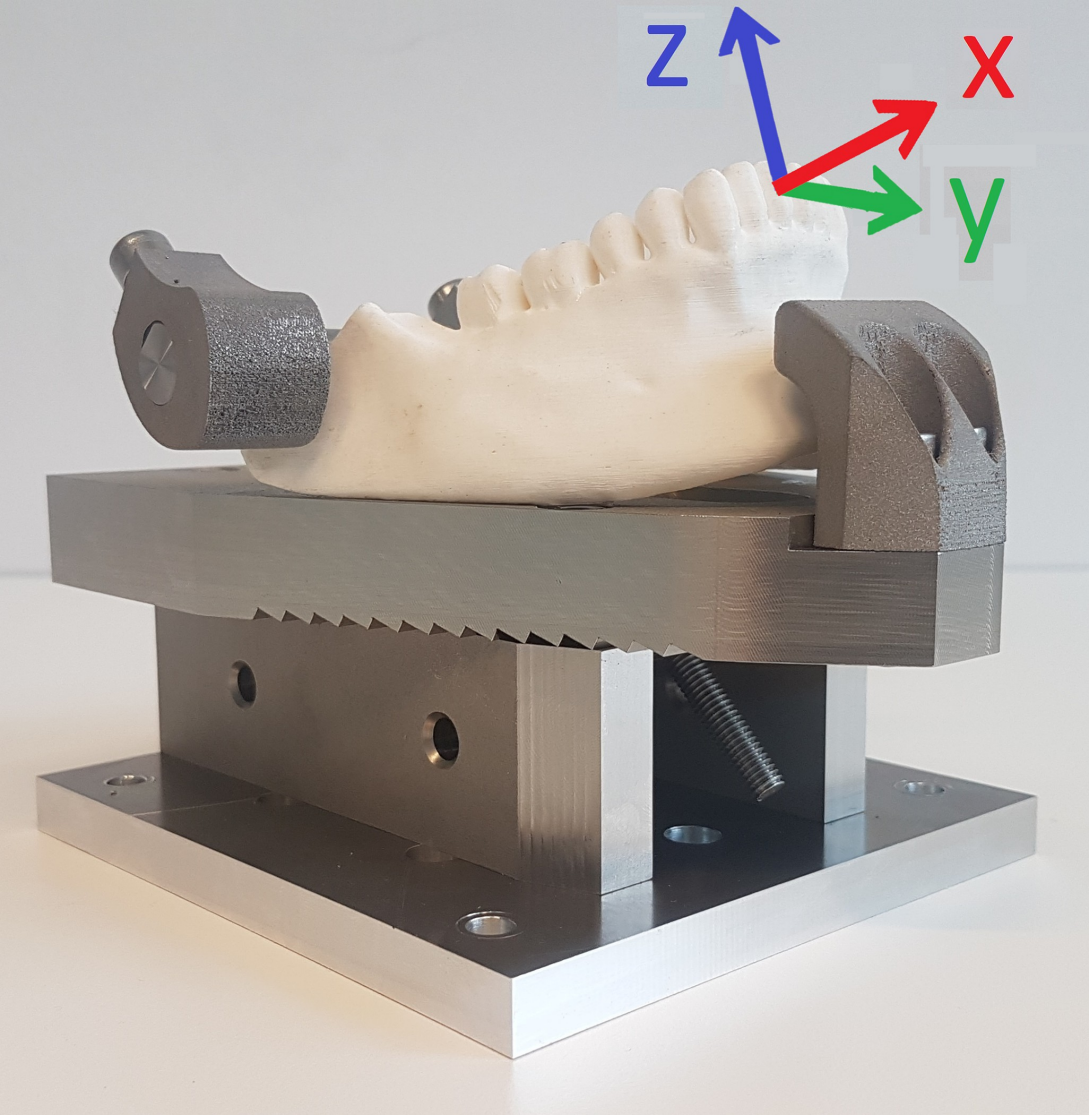
Lower jaw holding device. The white plastic model represents the reduced shape of the cadaveric lower jaw as prepared by the anatomical laboratorian. The reference frame is presented in 3 colored arrows. The X-axis (red arrow) represents the buccopalatal of buccolingual axis. The Y-axis (green arrow) represents the mesiodistal axis. The Z-axis (blue axis) represents the longitudinal axis.

Cadavers were selected by a single anatomy laboratorian based on the presence of multiple teeth and reduced to the necessary proportions by the first author to fit the measurement setup in the anatomical laboratory. This procedure has been described in detail in previous work [9]. A plastic model of the jaws in their reduced proportion is presented in Figs 1 and 2. Still in frozen condition, the lower jaw was reduced with an oblique cut using a reciprocating saw from the retromolar area to the gonial angle. The upper jaw was reduced by a horizontal cut at the infra-orbital level and a vertical cut behind the temporal root. Soft tissues were removed after defrosting with a scalpel, but care was taken not to remove any attached gingiva. The teeth itself and their directly surrounding hard and soft tissues were left intact with a wide margin as to ensure similar conditions throughout the experiments. To ensure clinically representative and generalizable results, 3 experienced oral and maxillofacial surgeons performed the procedures. They were instructed to perform tooth removal, as they would do in a regular clinical setting. Use of an elevator was not allowed, as the constantly changing position of the elevator relative to the tooth makes it unsuitable for our study goal. The ISO (International Standards Organization number 3950, Fédération Dentaire International) system was used as dental notation system.

### Measurement setup

**Fig 3:**
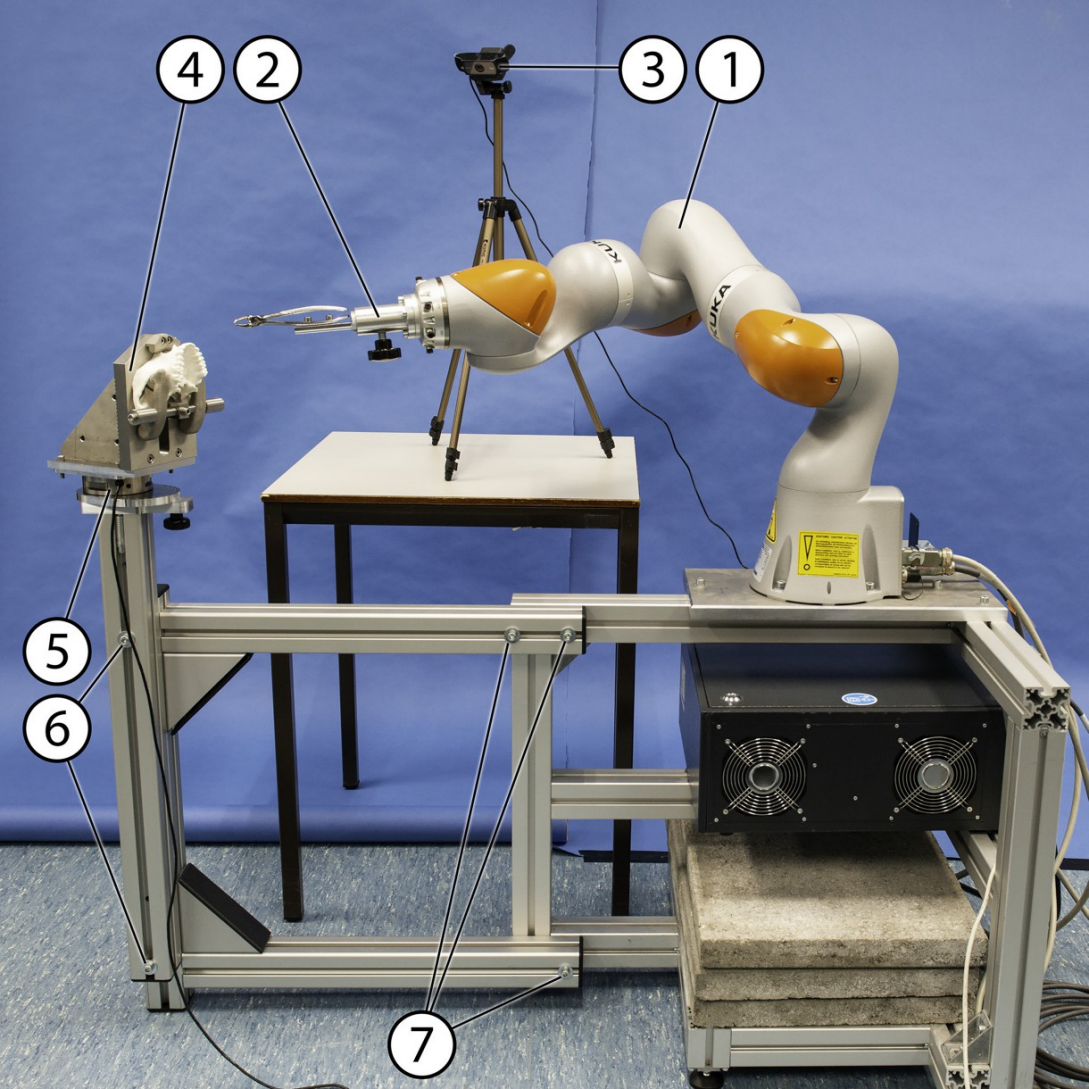
Overview of the setup. (1) passive robot arm (2) forceps holding device, (3) video camera, (4) upper jaw holding device (the lower jaw holding device not shown in this figure), (5) 6-axis force/torque sensor, (6) bolts to change vertical position, (7) bolts to change horizontal position.

An overview of the setup is presented in Fig 3. Summarizing, the main components, consisted of:

- a compliant robot arm (KUKA LBR iiwa 7 R800). It passively, compensated for gravity, followed the movements of the clinician at a frequency of 100 times per second (hertz, Hz). The robot arm also functioned as a calibration tool to allow for determination of the position and orientation of a tooth. Based on clinical experience and due to anatomical restrictions such as the shape of the alveolar process, the relative position of the roots to the cortical lining and the periodontal ligament rotational movements are considered significantly more important compared to translational movements (or displacement) [2,13]. This study, therefore, focused on rotational movements only.
- a 6-axis force/torque (FT) sensor (ATI industrial automation 16 bit Delta transducer) for registration of forces and torques in 3 dimensions at 20Hz.
- a video camera (Logitech C920 Pro HD) to record a video stream of the experiments.
- a custom-build and interchangeable upper and lower jaw-holding device (Figs 1 and 2).

The open-source framework Robot Operating System (ROS) was used for integration of all components. Metadata was added in a custom-build graphical user interface (GUI) to enable construction of a complete database for future analysis and to help to explain certain findings such as outliers. Metadata included, but was not limited to, the restorative and periodontal state of each tooth as these might influence the procedures in severe cases. Prior to each experiment, the surgeon was asked to determine the periodontal health by using a standard dental probe. States were divided into healthy (<3mm pocket depth), mild (3-5mm pocket depth) and severe periodontitis (>5mm pocket depth). Restorative states were classified into sound (no restorations), direct restorations (small <2 and large >2) and indirect restorations. The GUI also allowed for a direct post-experimental manual trim of the recorded data and the recording of any complications. The final few seconds of the procedures were excluded from the dataset. It is during this period where the tooth is taken out of its socket and extreme values are recorded, which should be considered as meaningless. The occurrence of any complications during the experiments were noted in the GUI. Experiments were classified as successful if the tooth was removed without complications or minor complications that did not influence the standard procedure. Experiments were classified as unsuccessful in case of serious issues, which might lead to unrepresentative results such as instant failure of indirect restorations or robot hardware/software issues. Visual inspection by the surgeon was performed after the procedure to determine complications which were sub-classified into boney wall fractures, root fractures or simple crown fractures (with removal of the root). Furthermore, after successful removal, anatomical features such as the root length and amount of roots were noted in the GUI. Because of the limited number of experiments in combination with the expected variability in this dataset, not all of the metadata was used in our analysis.

### Calibration and reference frame

The location and orientation of each tooth was calibrated before the start of an experiment. This step was essential to determine position and orientation of the tooth. It allowed for translation of the movements toward clinically relevant dimensions. The reference frames for all teeth in both upper and lower jaw were identical and can be found in Figs 1 and 2. The translation of movement data to a single reference frame enabled us to group ‘mirrored’ sets of teeth, for example the first premolars on the upper jaw (14 and 24) or lower second incisor (32 and 42). This was done so to create larger groups and to ease the interpretation of our results. For the calibration step, a straight periosteal elevator was mounted in the forceps-holding device and positioned parallel to the expected axis of each tooth and on top of the center of each crown. The flat part of the elevator pointed towards the lingual or palatal side. A mathematical translation was performed for each of the dental extraction forceps to align the axis according to the tooth frames (Table 1). The expected rotational center of the teeth was estimated 2mm below the center of the crown, as pointed out by the calibration tool.

**Table 1:** Tooth reference frame after mathematical translation.

Axis	Positive values	Negative values
Rotation around the bucco-palatal/lingual (X-)axis	Mesial angulation	Distal angulation
Rotation around the mesiodistal axis (Y-)axis	Buccoversion	Palatoversion / Linguoversion
Rotation around the longitudinal (Z-)axis	Mesiopalatal / Mesiolingual	Mesiobuccal

## Results

### Overview of experiments

A total of 127 experiments were performed on seven fresh-frozen Caucasian specimens. In 110 (86.6%) of these experiments full data was successfully recorded. The main reason (n = 8, 6.3%) for failure of experiments was insufficient fixation of the jaw causing displacement during the experiments, potentially leading to incorrect measurements. Other reasons were fracture of the teeth (n = 5, 3.9%), robot or software errors (n = 3, 2.4%) and excessive slippage of the forceps (n = 1, 0.8%). In the group of 110 successful experiments, most procedures happened without complications (n = 94, 85.5%). In other experiments, data was successfully recorded but minor complications were present of which a fracture of the boney wall was seen most frequently (n = 9, 8.2%). Most teeth had sound periodontal (n = 60, 54.5%) and restorative states (n = 45, 42.7%). For a complete overview of the basic characteristics, see Table 2.

**Table 2:** Base characteristics of experimental material and experiments.

Base Characteristics	Total number
**Fresh-frozen specimens** Upper jaws with teeth Lower jaws with teeth	**7** 6 6
**Total number of experiments** **Successful experiments**: Without complications Boney wall fracture Root fracture Crown fracture/failure (with root removal) **Unsuccessful experiments**: Insufficient fixation of jaw Crown fracture/failure (without root removal) Robot / software errors Excessive slippage of the forceps	**127** **110** 94 9 6 3 **17** 8 5 3 1
**Periodontal state (out of 110 experiments)** • healthy (pocket depth <3mm) • recessions • mild periodontitis (pocket depth 3-5mm) • severe periodontitis (pocket depth >5mm)	82 33 16 12
**Restorative state (out of 110 experiments)** • sound • direct restoration large (≥ 2 surfaces) • indirect restoration • direct restoration small (≤ 2 surfaces)	47 25 20 18

mm = millimeter.

### Axis dominance

**Fig 4:**
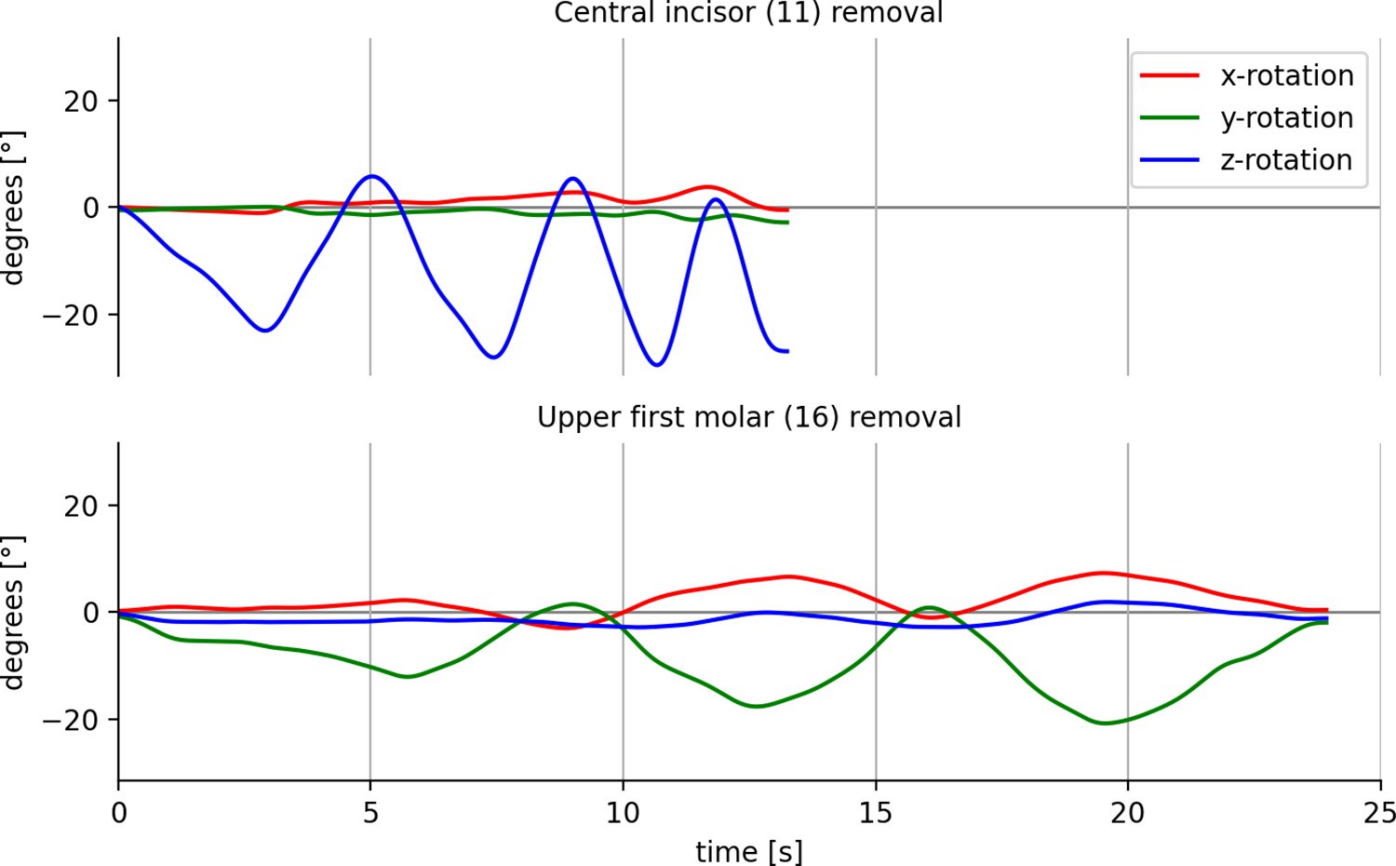
Visualization of movement data. Data recorded during removal of an upper central incisor (upper graph) and an upper first molar (lower graph). Movement around the longitudinal axis (blue line) was most dominant in the removal of a central upper incisor. Movement around the mesiodistal rotation (green line) was most dominant in removal of an upper first molar. In the upper first molar, rotation around the longitudinal axis was limited in contrast to the central incisor. X-rotation = rotation around the bucco-palatal or lingual axis. Y-rotation = rotation around the mesiodistal axis. Z-rotation = rotation around the longitudinal axis.

After translation of the data towards the same tooth frame (Table 1), movement data can be visualized as shown in Fig 4. It shows typical results of the removal of a central upper incisor and an upper first molar from the same cadaver jaw.

**Fig 5:**
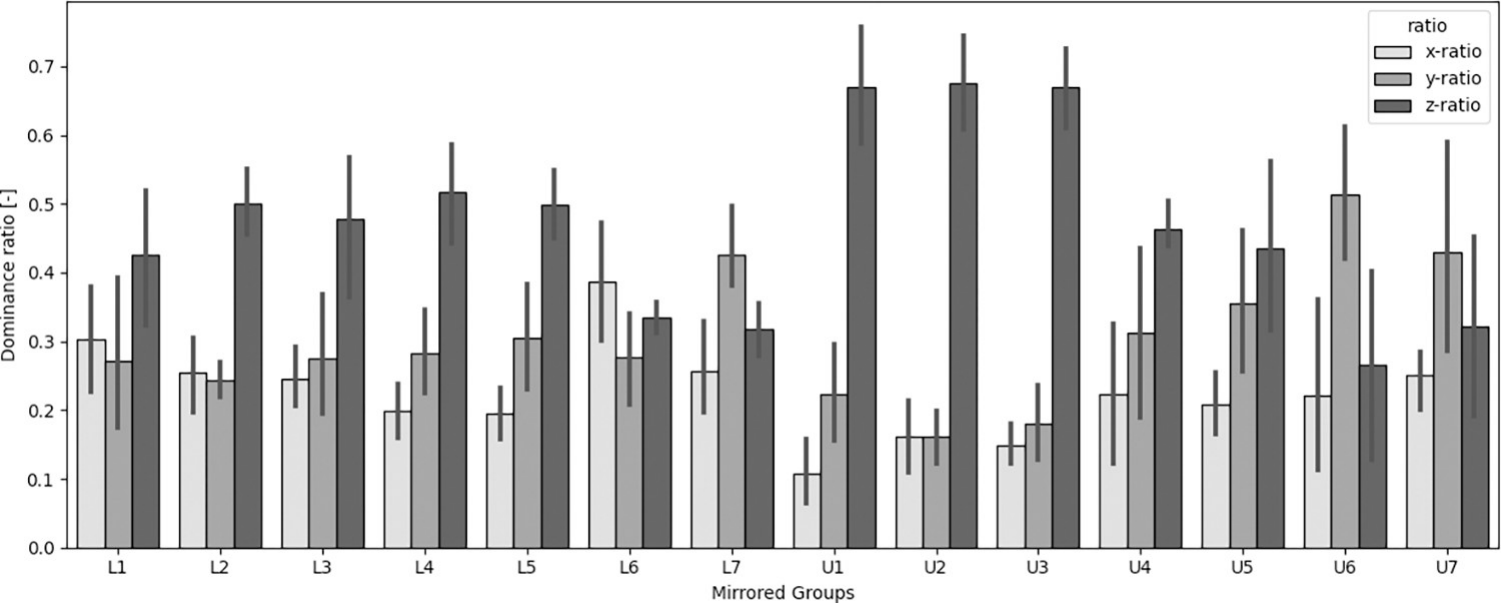
Axis dominance. Presented per ‘mirrored’ group of teeth and their standard deviation. The axis dominance was defined as the percentage of rotation that takes place around each specific axis relative to the other 2. X-ratio = rotation around the bucco-palatal or lingual axis. Y-ratio = rotation around the mesiodistal axis. Z-ratio = rotation around the longitudinal axis. L = lower. U = upper.

To determine along what axis most movement took place, the parameter ‘axis dominance’ was developed. It was calculated, or normalized, by dividing the line length of a single axis by the sum of the length of all 3 (Fig 4). The resulting parameter expresses the relative dominance in terms of amount of rotation along each axis. Results per group of teeth are shown in Fig 5. A clear dominance can be seen for rotations around the longitudinal axis of the tooth, especially in the upper frontal region. More dorsally, rotations around the mesial distal axis become more pronounced. Rotations around the buccopalatal or buccolingual axis (mesial/distal angulation) seem less dominant in all teeth.

### Range of motion and angular velocity

**Fig 6:**
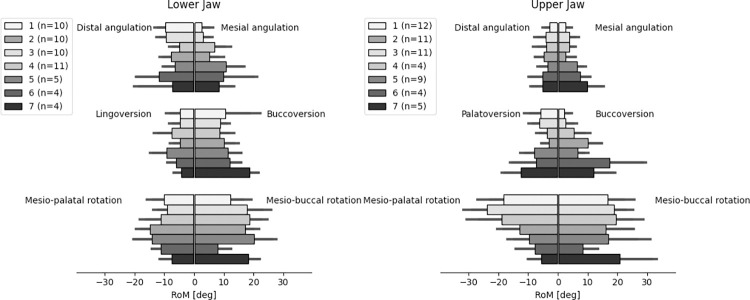
Range of motion. Averages of maximum rotations presented as a ‘range of motion’ along that axis. RoM = range of motion. deg = degree.

For each ‘mirrored’ group of teeth (i.e. the 14 and 24) maximum rotations as well as maximum angular velocities were calculated for all 6 directions separately. The averages of maximum rotations in both directions can be interpreted as a ‘range of motion’ along that axis and are presented in Fig 6. The largest range of motion can be seen in rotations around the longitudinal axis of the tooth, more so in the frontal region. Lowest range of motion was found in the direction of mesial and distal angulation, especially in the upper jaw.

**Fig 7:**
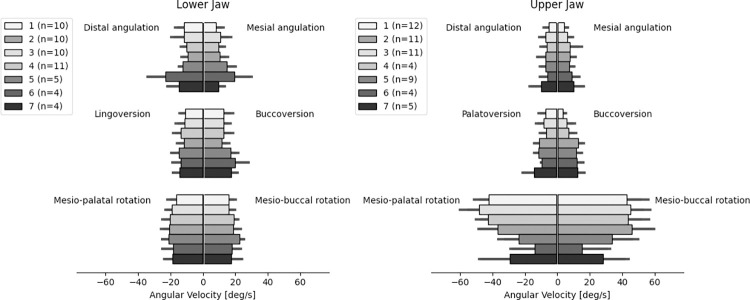
Angular velocity. Averages of maximum angular velocity in all clinically relevant dimensions. deg/s = degree per second.

The averages of the maximum angular velocity measured in each experiment per group of teeth are presented in Fig 7. Both in upper and lower jaw, highest angular velocities were seen in rotation around the longitudinal axis of the tooth, being more prominent in the upper jaw. This effect was less pronounced in the dorsal region of the upper jaw. A more constant angular velocity was detected across groups in the lower jaw.

## Discussion

The goal of this project was to capture movements of a clinician during successful tooth removal procedures in an accurate and reproducible manner. We hypothesized that this could be done through the use of robot technology. A descriptive analysis of experiments in an *ex vivo* setup was presented in clinically relevant dimensions. In total, 110 measurements on successful tooth removal procedures were included in this study.

In previous work, we reported on the forces and torques that were measured in all 6 directions during these procedures [8]. In this study, we focus on the direction, range and speed of movements.

For the ease of interpretation of this complex data, the parameter ‘axis dominance’ was developed. In this study, we found the most dominant axis of movement to be around the longitudinal axis of the tooth. It was also around this axis where the highest velocities were found and widest ranges of motion. More dorsally, movements along the buccopalatal or buccolingual axis became more relevant. Movement in mesial or distal angulation seemed less apparent, which is well in line with our clinical experience.

A useful comparison to previous literature is difficult since scientific data on this topic is, to our knowledge, non-existing. Most studies concerning analysis of tooth removal have focused on measuring forces and did so in a limited fashion [7]. Some studies distinguished between ‘twisting’ (around the tooth’s axis) and ‘rocking’ (buccolingual or buccopalatal) forces, indirectly describing some sort of movement [3,14,15]. What remains, for now, is a comparison of our results to our clinical experience. With rotations around the tooth axis being most prominent and buccopalatal/lingual movements being relatively more important in the dorsal area, this seems to correspond well.

A large standard deviation was found in our outcomes, showing significant variance in movements and velocity, even within groups of the same teeth. These findings might be expected, based on clinical experience and has several causes. Firstly, the extent of movement and its direction varies largely on both anatomic factors (i.e. root morphology, amount of roots, presence of adjacent teeth) and patient factors (i.e. bone morphology, mineral density) [14,16]. Although metadata was present that could partially explain some of this variance, due to the relative small sample size, no valuable correlation to these factors can be made at this point. Further research with a larger data sample could be valuable to determine any influences these factors have on tooth removal strategies. Another relevant factor that affects our results is the variety in surgeons. As experiments were performed by 3 different surgeons an increased variance in our results is to be expected. Especially since the surgeons did not receive any specific instructions or calibration, other than to remove teeth as they would do in a clinical setting. Finally, variance in our results might be caused by the calibration step, which is prone to error. Utmost care was taken to align the straight elevator correctly, but small deviations are inevitable as a significant part of the teeth is not visible in this phase and minor misjudgments might occur. Improvements to the calibration step, for example using imaging data and single registration of the jaw instead of individual teeth are necessary in future projects.

The outcomes of this study should be carefully interpreted. Mainly, because it is unknown in what way fresh-frozen cadavers relate to the clinical situation and because of the small data sample. We aimed for 100 successfully recorded procedures to enable a first and representative analysis. It can be concluded that our results should be regarded as a first presentation of the right order of magnitude when considering movements and velocities in tooth removal. A confirmation of the data in a larger sample is necessary.

Some disadvantages of the setup should be discussed. To minimize any restrictions of the robot arm in terms of movement, besides compensation for gravity, an optimal starting position for upper and lower jaw was determined in which the joints were least likely to reach a ‘joint limit’. When a ‘joint limit’ of the robot is reached, it needs to move other joints to facilitate further movement in a specific direction. This could deliver some resistance, which might prevent the surgeon moving in a specific direction and therefore influencing the movement pattern. With the use of predefined optimal starting positions, these restricted movements were prevented as much as possible, but a minimal effect might be present. Despite this issue, feedback from the surgeons was positive regarding the clinical representativeness of their removal strategies. Another disadvantage is the use of dental forceps over elevators, which are frequently used in clinics. Due to the constantly changing position relative to the tooth, the use of elevators was excluded from this study.

Future work should focus on improving the measurement setup first, especially regarding the calibration step, which can be considered as cumbersome and potentially lead to calibration errors. This could be overcome by the use of registration of image data, obtained prior to the experiments (CT-imaging). After that, the database should be extended to evaluate in which manner clinical features influence tooth removal strategies. Preferably this data is gathered in an *in vivo* research setting, but this is considered very challenging [9]. The data gathered in this and future work can be used to improve dental education in tooth removal in an evidence-based manner [17].

To the authors’ best knowledge, this is the first time that different aspects of motion in tooth removal have been measured and analysed. We hypothesized that this could be done through the use of robot technology. Despite the mentioned shortcomings of this innovative work, the data presented here seems to define some order of magnitude when considering range of motion and angular velocities during tooth removal. We are convinced that we have gained important first scientific insights into tooth removal procedures and that robot technology was essential in doing so. The current database is, however, limited and its extension is essential to confirm our results in future research. An extensive dataset can be used to find clinically relevant factors influencing our proposed parameters. Finally, improved understanding of these complex procedures can be used to improve educational tools in an evidence-based manner [17,18].
